# Donor-specific pathological features associate with genetic background, lesion type distribution, and clinical heterogeneity in multiple sclerosis

**DOI:** 10.1007/s00401-026-03040-3

**Published:** 2026-06-25

**Authors:** Lukas Lütje, J. Q. Alida Chen, Jörg Hamann, Joost Smolders, Inge Huitinga, Aletta M. R. van den Bosch

**Affiliations:** 1 Neuroimmunology Research Group, Netherlands Institute for Neuroscience, Amsterdam, 1105 BA The Netherlands; 2 Department of Experimental Immunology, Amsterdam Institute for Immunology and Infectious Diseases, Amsterdam University Medical Center, Amsterdam, 1105 AZ The Netherlands; 3 Departments of Neurology and Immunology, MS Center ErasMS, Erasmus Medical Center, University Medical Center, Rotterdam, 3015GD The Netherlands; 4 Center for Neuroscience Swammerdam Institute for Life Sciences, University of Amsterdam, Amsterdam, 1090 GE The Netherlands; 5 Department of Experimental Neuropathology, IRCCS Humanitas Research Hospital, 20089 Milan, Italy

**Keywords:** Multiple sclerosis, Neuropathology, Microglia nodules, Broad rim lesions, Remyelination, Perivascular cuffs

## Abstract

Multiple sclerosis (MS) shows pronounced pathological and clinical variability between individuals, reflecting differences in genetic susceptibility, inflammatory activity, and tissue repair. This variability complicates efforts to relate lesion pathology to clinical trajectories. In previous work in the Netherlands Brain Bank MS autopsy cohort (NBB-MS), we showed that relative proportions of different lesion types, lesion load, and microglia/macrophage activity score, associate with clinical severity, while also revealing marked inter-individual variability. Here, we extend these observations by examining whether selected donor-specific pathological features relate to genetic background, quantitative lesion type distributions, and clinical disease course, and thereby help contextualize this heterogeneity.

Brain tissue from 287 NBB-MS donors was assessed for the presence of the donor-specific pathological features, namely perivascular cuffs, microglial nodules, broad rim lesions (BRLs), and remyelination efficiency. Perivascular cuffs and microglial nodules were more prevalent among carriers of the MS susceptibility allele HLA-DRB1*15:01 (rs3135388). BRLs and perivascular cuffs were enriched in carriers of the MS severity-associated SNP in the *DYSF–ZNF638* locus (rs10191329). Perivascular cuffs associated with increased microglia/macrophage activation score and decreased age at death. Microglial nodules in the normal appearing white matter associated with a higher proportion of active lesions. BRLs were linked to increased proportions of active and mixed active/inactive lesions, higher brainstem lesion rate, and a higher age related MS severity score. Poor remyelination efficiency associated with a higher proportion of mixed active/inactive and inactive lesions, and a shorter disease duration.

Together, these findings show that specific pathological features of donors relate to genetic risk, lesion type distribution, and clinical outcome. Integrating these donor-specific pathological features alongside lesion classification will enable a more biologically refined interpretation of post-mortem MS tissue study results and will improve understanding of inter-individual heterogeneity in MS.

**Supplementary Information:**

The online version contains supplementary material available at 10.1007/s00401-026-03040-3.

## Introduction

Multiple sclerosis (MS) is a chronic inflammatory disease of the central nervous system (CNS), with focal demyelinating lesions that vary widely in their inflammatory activity, cellular composition, and capacity for repair [2, 7, 24, 29]. People with MS vary in genetic susceptibility, lesion pathology, and clinical course, underscoring substantial inter-individual biological heterogeneity [15, 29]. Understanding the pathological correlates of this heterogeneity remains a major challenge, particularly when interpreting post-mortem tissue and relating neuropathological findings to disease progression.

Standardized autopsy procedures and lesion classification systems enable quantitative assessment of MS lesion burden and lesion type distributions across the CNS [27, 29]. Using these approaches, we and others previously showed that inflammatory lesion activity often persists until death and that inflammation closely correlates with neurodegeneration [17, 29, 32]. A higher lesion load, an increased proportion of mixed active/inactive (mixed) lesions, and perivenular inflammation further associate with a faster disability accumulation [29, 32]. Importantly, these analyses also revealed substantial variability in lesion burden and relative proportions of different lesion types in the white matter (i.e. active, inactive, remyelinated, mixed) and in the cortex between donors [26, 33]. This variability suggests that, beyond lesion type classification itself, donor-specific biological differences influence how lesions form, evolve, and repair during the disease course.

Specific pathological features of donors may reflect persistent and dominant inflammatory and reparative features [28]. These include perivascular cuffs, which consist of accumulations of lymphocytes (B and T cells) in the perivascular space [9, 14, 16, 39], small HLA-DR^+^ microglia clusters, or nodules, in the normal-appearing white matter [10, 42], mixed lesions with a broad rim of HLA-DR^+^ myeloid cells (broad rim lesions, BRLs) [25], and the proportion of remyelinated lesions reflecting donor-specific repair capacity [6, 7]. Each of these features could indicate inter-individual differences in pathological processes, which together could provide complementary information that helps explain why lesion patterns and clinical disease course differ between individuals.

Here, we investigated whether recently described donor-specific pathological features, defined by cuffs, nodules, BRLs, and remyelination efficiency, reflect relevant biological processes associated with genetics, lesion patterns, and clinical disease course. Using a large, well-characterized Netherlands Brain Bank MS autopsy cohort (NBB-MS), we show that these features capture substantial inter-individual heterogeneity in MS.

## Materials & Methods

### Human tissue and ethics statement

The procedure for brain donation to the NBB and the use of clinical and pathological information for research were approved by the medical ethics committee of the VU medical center (Amsterdam, The Netherlands, approval number 2009/148). Written informed consent for brain autopsy and the use of material and clinical data for research purposes was obtained from all donors in accordance with national ethical guidelines.

This study included 287 donors with MS from the NBB autopsy cohort collected between 1990–2021. MS pathology was confirmed by a certified neuropathologist. Donors with relevant neurological or systemic comorbidities were excluded from analyses. Only donors with Braak scores ≤ 3 were included in the analyses.

### Tissue sampling and histopathological staining

Post-mortem brain tissue was dissected from standardized locations in the brainstem (BRS). MS lesions were identified macroscopically and/or guided by post-mortem magnetic resonance imaging. On average 21.8 (± 11.0) tissue blocks and 35.4 (± 31.3) lesions were dissected per donor. Tissue blocks were formalin-fixed paraffin-embedded (FFPE).

At the NBB, sections from all FFPE tissue blocks were double-immunostained for human leukocyte antigen (HLA-DP-DR-DQ, hereafter referred to as HLA; M0775; DAKO, Glostrup, Denmark, with 3,3′-diaminobenzidine (DAB)-nickel) and proteolipid protein (PLP; MCA839G; Serotec, Oxford, UK, with DAB) to visualize HLA^+^ activated microglia/macrophages and myelin, respectively, as described previously [27, 29]. Ferrous and ferric iron was visualized with the DAB-enhanced Turnbull Blue (TBB) staining method [31].

### MS lesion classification

Histopathological characterization of white matter, deep grey matter, and cortical grey matter lesions was performed by the NBB according to the Kuhlmann et al*.* and Van der Valk et al*.* classification system, previously utilized by Luchetti et al*.* [27, 29, 44]. In short, white matter and deep grey matter lesions were characterized based on presence of innate inflammatory activity as assessed by HLA^+^ microglia/macrophages, and myelin status, assessed by PLP staining. Lesions were categorized as 1) reactive sites, defined by aggregates of HLA^+^ microglia/macrophages without demyelination, 2) active lesions, characterized by dense accumulation of HLA^+^ microglia/macrophages throughout the lesion and partial loss of myelination, 3) mixed lesions (also referred to as chronic active lesions), displaying a rim of HLA^+^ microglia/macrophages surrounding a fully demyelinated and hypocellular center, 4) inactive lesions, showing hypocellularity and demyelination throughout the lesion, and 5) remyelinated lesions exhibiting sparse HLA^+^ microglia/macrophages and partial myelination. Cortical grey matter lesions were characterized based on their anatomical location as 1) leukocortical, 2) intracortical, and 3) subpial lesions [20, 29].

### Donor stratification based on pathological features

Donors were stratified based on the presence or absence of donor-specific pathological features that reflect distinct inflammatory and reparative states, following HLA-PLP immunohistochemistry: 1) perivascular cuffs [9, 14, 16, 39], defined as the presence of at least one perivascular space with an exaggerated accumulation of lymphocytes in any examined tissue block [14], 2) microglial nodules [43], defined as the presence of at least one cluster of ≥ 4 ramified microglia in the examined tissue blocks, 3) broad rim lesions (BRLs), defined by at least one mixed lesion with a hypercellular HLA^+^ rim with average width of ≥ 1 mm surrounding a hypocellular lesion core [25], and 4) remyelinating efficiency, classifying donors as efficiently remyelinating donors (ERDs) or poorly remyelinating donors (PRDs) [6, 7], based on a remyelinated lesion proportion of ≥ 0.27 and < 0.27, respectively. This threshold corresponds to the median remyelinated lesion proportion of the NBB MS autopsy cohort (239 donors, 1990–2020) [7]. In addition, donors lacking all features described in 1–4 were analyzed as a separate group, and as cohort validation, donors were stratified based on the presence of active lesions.

### Genetic analysis

Two clinically relevant single nucleotide polymorphisms (SNPs) were analyzed: 1) rs3135388, a tagging (tag)-SNP for the MS susceptibility allele HLA-DRB1*15:01 [34] and 2) rs10191329, located within the *DYSF–ZNF638* locus and previously associated with MS severity [22]. Genotyping for rs10191329 was performed using the KASP genotyping platform (LGC Genomics). Genotype data for rs3135388 were obtained from Illumina SNP-array analyses.

### Detailed lesion assessment

The brainstem lesion rate was calculated as the sum of active, mixed, inactive, and remyelinated lesions identified in BRS divided by the number of BRS tissue blocks dissected, to ensure standardized and normalized anatomical sampling across donors. The cortical lesion rate was calculated as the total number of all leukocortical, intracortical and subpial lesions across all tissue blocks divided by the number of tissue blocks with cortex present [22]. Proportions of active, mixed, and inactive lesions were calculated relative to the total number of all active, mixed, inactive, and remyelinated lesions. The proportion of remyelinated lesions was calculated relative to the combined number of inactive and remyelinated lesions.

Microglia/macrophage activation was quantified using the microglia/macrophage activation score (MMAS) in active lesions and mixed lesions, based on cell morphology as described previously [29]: 0 = thin and ramified, 0.5 = amoeboid with few ramifications, 1 = foamy. The MMAS represents the average score across evaluated lesions.

Previously defined neuropathological dimensions were calculated to capture shared variance across multiple pathological parameters, including MS lesion rate and lesion load, lesion type proportions, microglia/macrophage morphology, and presence of nodules and cuffs, as described by de Boer et al*.* [9]. Briefly, dimension 1 reflects active demyelination and inflammatory lesion activity, dimension 2 reflects microglia/macrophage activation without demyelination, possibly reflecting lesion onset, and dimension 3 reflects chronic lesion scarring and gliosis. Even though cuffs, nodules, and remyelinated lesion proportions, together with other pathological features, have been used as input for the identification of pathological dimensions, this study investigated how isolated donor-specific pathological features were reflected in the collective pathological dimensions.

### Clinical parameters

Clinical data were obtained from medical summaries provided by the NBB. Assessed parameters included age at symptom onset, age at death, and total disease duration. Disability progression during the disease course was assessed using the Kurtzke’s Expanded Disability Status Scale (EDSS). Age at EDSS6 was used to calculate the age related MS severity score (ARMSS) [30]. The clinical parameters are summarized in Table 1. Brain weight, post-mortem delay, and cerebrospinal fluid pH were evenly distributed between subgroups in all comparisons.

**Table 1:** Clinical and demographic characteristics of the MS cohort

	All MS donors	Cuffing		Nodules		Broad rim lesions		Remyelination		Any one of four pathological features		
		Cuffs	No cuffs	Nodules	No nodules	BRLs	No BRLs	PRDs	ERDs	Present	Absent	*P*-values
Donors (n)	287	64	187	182	69	52	165	111	129	175	29	
Sex, female (%)^a^	65.9%	68.3%	65.2%	70.2%^#^	55.1%^#^	63.4%	68.5%	65.8%	66.4%	68.0%	62.0%	#
pH of CSF^b^	6.5 (0.3)	6.5 (0.3)	6.5 (0.3)	6.5 (0.3)	6.5 (0.4)	6.4 (0.3)	6.5 (0.3)	6.5 (0.4)	6.5 (0.3)	6.5 (0.3)	6.5 (0.3)	n.s
PMD (hrs:min)^**b**^	08:44 (04:48)	08:02 (02:16)	8:49 (4:11)	08:23 (02:41)	09:15 (05:47)	08:36 (01:59)	08:11 (02:26)	08:37 (04:57)	08:48 (02:32)	08:15 (02:17)	09:09 (2:22)	n.s
Brain weight (g)^b^	1194.6 (132.1)	1184.3 (145.2)	1199.1 (127.5)	1189.4 (132.8)	1212.1 (129.4)	1203.1 (126.1)	1185.5 (130.3)	1187.4 (128.8)	1195.2 (133.4)	1183.5 (127.0)	1214.6 (126.5)	n.s

Data are presented as the mean (standard deviation), unless otherwise indicated. Any one of four pathological features indicates donors with presence of cuffs, nodules, BRLs and/or poor remyelination, or absence of cuffs, nodules, BRLs and efficient remyelination. ^a^Pearson’s Chi-squared test, ^b^Student’s t-test. ^#^Significant difference between donors with and without nodules (*P* = 0.036). *MS* multiple sclerosis; *BRL* broad rim lesion; *PRDs* poorly remyelinating donors; *ERDs* efficiently remyelinating donors; *CSF* cerebrospinal fluid; *PMD* post-mortem delay; *n.s* not significant

### Data availability

An overview of pathological, clinical, and genetic characteristics of the MS donors included in this study is provided by the Netherlands Neurogenomics Database (NND, https://nnd.academy/). Any data inquiries will be honored upon reasonable request.

### Statistical analyses

All discrete and continuous data were analyzed using Student’s t-test. Proportional data were analyzed using the Wilcoxon rank-sum test. *P*-values were corrected for multiple testing using false discovery rate (FDR) correction, with corrected *p*-values < 0.05 considered statistically significant (stats package, v4.4.3). Genetic associations were assessed using Fisher’s exact test and categorical data of clinical parameters were assessed using the Pearson’s chi-squared test. Here, p-values < 0.05 were considered significant (stats package, v4.4.3). All analyses were performed using R (version 4.4.3).

## Results

### Overview of MS lesion heterogeneity and donor-specific pathological features

**Fig. 1:**
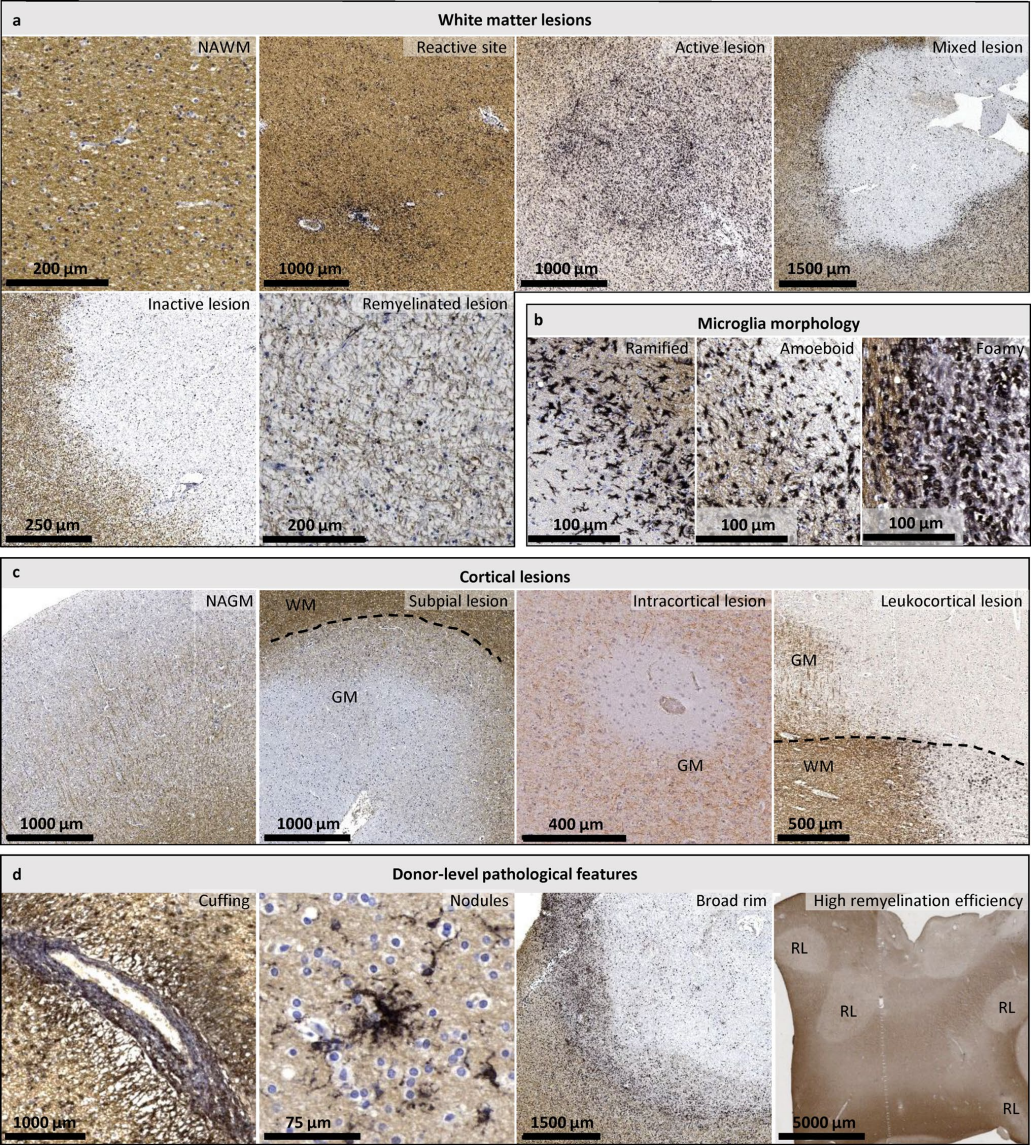
Representative images of common features in MS pathology and donor-specific pathological features. All images are stained for HLA (black) and PLP (brown). **a** Normal-appearing white matter and white matter lesions characterized in MS tissue of the NBB. **b** Microglia/macrophages expressing HLA with distinct morphologies at the edge of mixed active/inactive lesions. **c** Grey matter with and without lesions. **d** Donor-specific pathological features include perivascular cuffs, nodules of ≥ 4 HLA^+^ microglia/macrophages surrounded by intact myelin, mixed lesions with broad rim and high remyelination efficiency. *GM* grey matter; *NAGM* normal-appearing grey matter; *NAWM* normal-appearing white matter; *RL* remyelinated lesion; *WM* white matter

To address inter-donor heterogeneity in MS, we investigated whether donor-specific pathological features reflect underlying biological mechanisms, which associate with genetic background, relative lesion type abundance, and clinical disease course. Donors were stratified according to four donor-specific pathological features: perivascular cuffs, microglial nodules, broad rim lesions (BRLs), and remyelination efficiency. Representative images of these pathological features, as well as lesion types observed and characterized within the NBB-MS autopsy cohort, are shown in Fig. 1. Additional lesion morphologies reflecting transitional, confluent, variable remyelinated states, and iron content, were observed within the cohort (Suppl. Fig 1). These underscore that MS pathology exists along continua rather than discrete categories, as well as indicate that residual heterogeneity remains beyond categorical lesion states.

Across white matter regions, including subcortical white matter, brainstem, and spinal cord, normal-appearing white matter, reactive sites, active lesions, mixed lesions, inactive lesions, and remyelinated lesions were identified (Fig. 1a). Previously, presence of active lesions and a higher proportion of mixed lesions have been linked to a worse clinical disease course [29, 32]. Active and mixed lesions further differed in microglia/macrophage morphology, ranging from ramified to amoeboid and foamy phenotypes (Fig. 1b). In the cortex, subpial, intracortical and leukocortical were identified (Fig. 1c). Additional pathological features were identified in some donors, but not in others (Fig. 1d). In the white matter, perivascular cuffs were observed in 25.5% (Table 1; 64 of 251 donors) of donors, and microglial nodules were observed in 72.5% (Table 1; 182 of 251 donors). Mixed lesions vary in rim thickness, allowing distinction between classical lesions and BRLs, which were identified in 24.0% of donors (Table 1; 52 of 217 donors) [25]. The extent of remyelination further differed between people with MS, as the number of remyelinated lesions can be found in varying degrees within MS donors (Fig. 1d). Based on remyelinated lesion proportions, donors were classified as poorly remyelinating donors (PRDs; 46.25%; 111 of 240 donors) or efficiently remyelinating donors (ERDs; 53.75%; 129 of 240 donors).

**Fig. 2:**
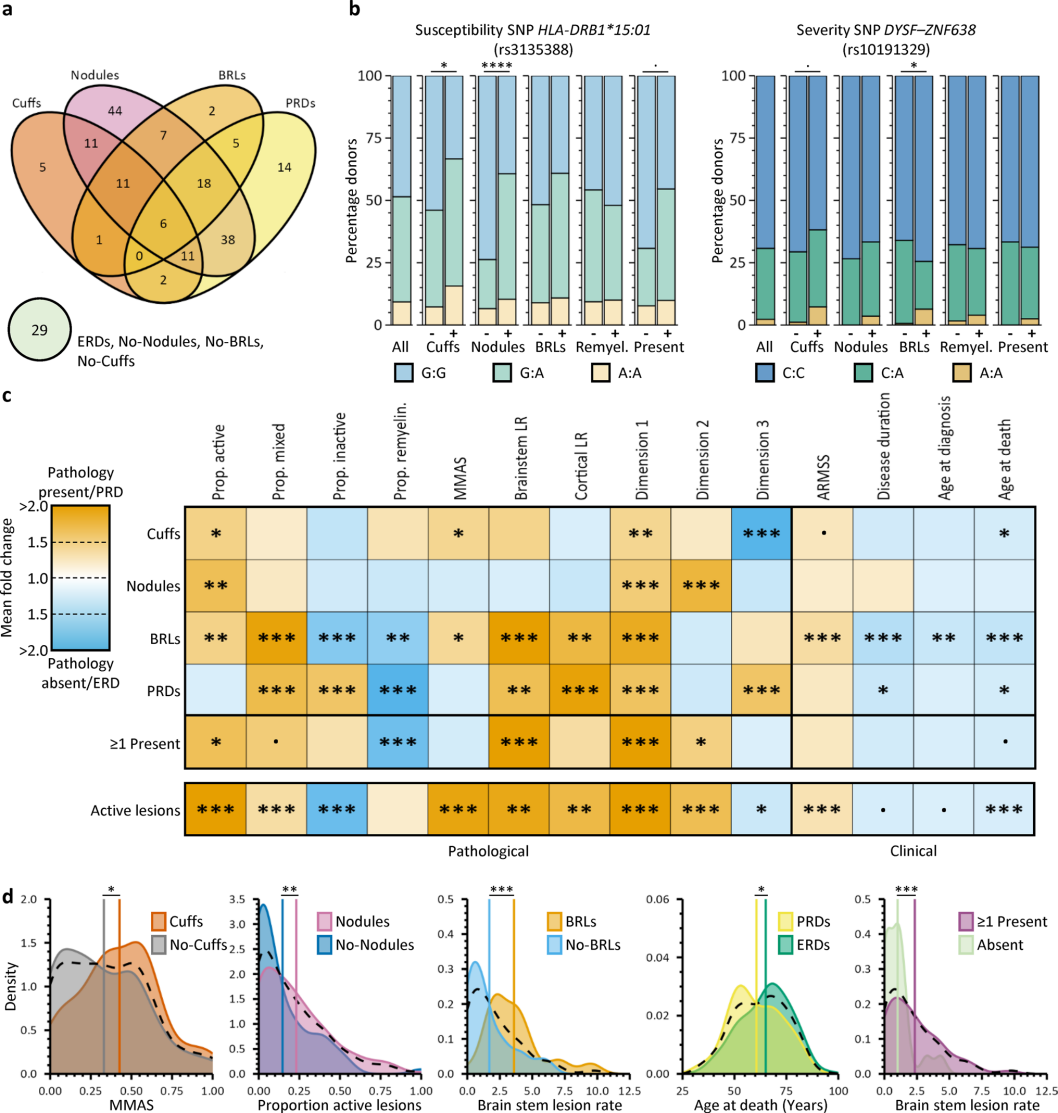
Comparison of clinical and pathological features between distinct cohorts. **a** Venn diagram displaying the number of donors having nodules, BRLs, poor remyelination capacity, and/or cuffs. Only donors with available information for all four pathological features are shown. **b** Genotype distribution of susceptibility SNP HLA-DRB1*15:01 (rs3135388) and progression SNP *DYSF–ZNF638* (rs10191329) in the NBB-MS and stratified cohorts. **c** Pathological and clinical comparisons of the NBB-MS cohort stratified for the presence of nodules, BRLs, remyelination capabilities, cuffs and ERDs without any donor-specific pathologies. The legend indicates whether group means are higher in donors with (orange) or without (blue) the respective pathology; hue indicates fold difference. **d** Density plots showing microglia/macrophage activation score for cuffs, proportion of active lesions for nodules, brainstem lesion rate for BRLs and for donors with any pathology present, and age at death for remyelination efficiency. The dashed black line represents the average of NBB-MS cohort. •*P* < 0.1, *P < 0.05, **P < 0.01, ***P < 0.001. *ARMSS* age related MS severity score; *BRL* broad rim lesion; *Dim* dimension; *ERDs* efficiently remyelinating donors; *LR* lesion rate; *MMAS* microglia/macrophage activation score; *PRD* poorly remyelinating donors

These features frequently co-occurred, particularly BRLs with perivascular cuffs and nodules with PRDs (Fig. 2a). Only a minority of donors (29 of 204; 14.2%) lacked perivascular cuffs, microglial nodules, BRLs, and were classified as ERDs. Five of these donors had previously been clinically characterized as very slowly progressing MS patients, whereas none were classified as rapidly progressing [25]. This overlap supports the concept that these features reflect intersecting biological processes rather than mutually exclusive disease categories.

### Genetic associations with donor-specific pathological features

Given the established contribution of genetic variation to MS susceptibility and severity, we examined whether donor-specific pathological features associate with clinically relevant MS-associated genetic variants [15, 22, 34] (Fig. 2b). Homozygous carriership of the MS susceptibility allele HLA-DRB1*15:01 (tag-SNP rs3135388) [34] increases the risk of developing MS 3–6 fold [40]. Previously, HLA-DRB1*15:01 has been associated with higher extent of inflammation and demyelination in the motor cortex and spinal cord [11, 45]. We extend on these findings by demonstrating that homozygous A carriers of HLA-DRB1*15:01 were significantly more likely to exhibit perivascular cuffs and microglial nodules at autopsy. These associations align with the role of HLA-DRB1*15:01 in shaping adaptive immune responses and support the involvement of lymphocyte recruitment and early innate immune activation in genetically susceptible individuals.

We next examined the MS severity-associated tag-SNP rs10191329 within the *DYSF–ZNF638* locus, of which homozygous A carriers reached walking-aid dependence 3.7 years earlier [13, 22]. Donors with perivascular cuffs showed a trend (*P* = 0.050) toward enrichment of this genotype. While genetic distribution of donors with or without microglial nodules did not differ significantly, all homozygous risk allele carriers exhibited nodules. The enrichment of carriers in donors with BRLs reported by Klotz & Smolders et al. remained significant with inclusion of additional donors [25]. No genotype differences were observed between rs10191329 and remyelination efficiency. Importantly, all donors carrying the homozygous risk genotype displayed at least one donor-specific pathological feature, linking genetic severity risk to pathological burden.

### Donor-specific pathological features associate with lesion type proportions and neuropathological dimensions

We explored how donor-specific pathological features relate to lesion (sub)type proportions, MMAS score, BRS and cortical lesion rate, and previously defined neuropathological dimensions [25] (Fig. 2c and d; Suppl. Fig 2; Suppl. Table 1). Neuropathological dimensions were defined as continuous axes capturing shared variance across pathological parameters: dimension 1 reflects active demyelination and inflammatory activity, dimension 2 captures microglial activation, and dimension 3 represents chronic lesion scarring and gliosis [9].

To validate the cohort, donors were stratified for presence of active lesions. As expected [29, 32], donors with active lesions showed a more severe clinical disease course and higher proportion of mixed lesions, an elevated MMAS, and an increased BRS and cortical lesion rate. Donors with active lesions also scored higher on dimensions 1 and 2, and lower on dimension 3, consistent with increased demyelination and microglial activity (Fig. 2c).

Donors with perivascular cuffs exhibited increased proportion of active lesions, increased MMAS (Fig. 2d), and scored higher on dimension 1 reflecting active demyelination and inflammatory lesion activity, alongside lower scores on dimension 3 reflecting chronic scarring. This profile is consistent with sustained inflammatory lesion activity. Donors with microglial nodules showed a higher proportion of active lesions (Fig. 2d), and a higher score on dimensions 1 and 2, reflecting enhanced microglial activation. Other lesion type proportions, MMAS, BRS lesion rate, and cortical lesion rate were comparable between donors with and without nodules. Donors with BRLs displayed a distinct pathological signature characterized by increased proportions of active and mixed lesions, reduced proportions of inactive and remyelinated lesions, elevated MMAS, higher BRS lesion rate (Fig. 2d) and higher cortical lesion rate. These donors scored higher on dimension 1, reflecting ongoing inflammation and demyelination. Compared to ERDs, PRDs exhibited higher proportions of mixed and inactive lesions, increased BRS- and cortical lesion rate, and elevated scores on dimension 1 and 3, reflecting inflammatory activity and increased scarring. Donors without donor-specific pathological features (ERDs lacking nodules, BRLs and perivascular cuffs) showed lower BRS lesion rate (Fig. 2d), lower proportion of active lesions, and higher proportions of remyelinated lesions, and lower scores on dimensions 1 and 2, reflecting reduced inflammation and demyelination and reduced scar formation. Together, these patterns suggest that donor-specific pathological features reflect overlapping but distinct biological processes that shape lesion dynamics and overall pathological burden, thereby contributing to MS heterogeneity.

### Donor-specific pathological features associate with clinical disease course

To assess clinical relevance of donor-specific pathological features, we compared the sex, age at diagnosis, disease duration, age related MS severity score (ARMSS), and age at death between donor groups (Table 1; Fig. 2c; Suppl. Fig 3; Suppl. Table 1).

Donors with perivascular cuffs died at a younger age and showed a trend toward higher ARMSS (*P* = 0.073). Presence of microglial nodules was more frequently observed in females rather than males (*P* = 0.036) (Table 1), but donors with or without nodules did not show differences in clinical disease course. Consistent with the earlier publication, donors with BRLs exhibited a more severe clinical course, characterized by higher ARMSS, shorter disease duration, and younger age at death [25]. Additionally, we found that donors with BRLs displayed their first symptoms at a younger age compared to donors without BRLs. PRDs displayed shorter disease duration and younger age at death compared to ERDs (Fig. 2d). Lastly, donors lacking all four pathological features tended to die at an older age, although this did not reach statistical significance (*P* = 0.057). These findings indicate that donor-specific pathological features correspond to clinically relevant variation in MS disease course, with BRLs and remyelination efficiency showing the strongest associations with disability accrual and survival.

## Discussion

MS is characterized by pronounced inter-individual heterogeneity. MS lesion characterization has demonstrated substantial variability in lesion burden, composition, and inflammatory activity between and within donors, and previous studies by us and others have linked this variability to differences in clinical progression [18, 27, 29, 32]. However, the biological mechanisms of this heterogeneity between individuals still remain incompletely understood.

In this study, we extend these previous MS lesion-focused analyses by examining whether donor-specific pathological features (perivascular cuffs, microglial nodules, BRLs, and remyelination efficiency) associate with inter-individual differences in lesion patterns and disease course in a large MS autopsy cohort of the NBB. We show that these features reflect inter-individual differences in key pathological processes that are linked to genetic background, relate to distinct relative lesion type abundance and neuropathological dimensions and associate with clinically relevant differences in disease course.

Perivascular cuffs have been associated with lesion formation and demyelinating lesion activity in MS [9, 14, 37, 39], and with a more severe clinical disease trajectory [14, 32]. Our findings suggest that the presence of perivascular cuffs are genetically influenced, as they are enriched among carriers of variants associated with both MS susceptibility and disease severity. This is consistent with a role for perivascular cuffs in both lesion initiation and sustained inflammatory activity. Pathologically, this feature is associated with increased predominance of foamy microglia/macrophages in active and mixed lesions, which is consistent with ongoing myelin phagocytosis [4, 29, 41, 42, 47]. This inflammatory profile is further reflected in neuropathological dimensions indicative of ongoing demyelinating activity with limited progression toward chronic scarring. Our data align with previous research [14, 32] linking perivascular cuffs to clinical severity. Overall, these findings support the interpretation of perivascular cuffs as a marker of sustained inflammatory activity across the disease course.

Microglial nodules have been proposed to represent early inflammatory events and possible starting points of MS lesions [9, 43]. Their enrichment among carriers of the HLA-DRB1*15:01 susceptibility-associated allele supports a link between genetic risk and early innate immune activation within the CNS, and their sex bias parallels known sex differences in MS susceptibility [8]. The association of nodules with increased proportions of active lesions further supports their role in early lesion dynamics [3, 9, 43]. Despite the genetic and pathological associations with nodules, their presence was not associated with clinical course. Together, these findings are consistent with the interpretation that the microglial nodules reflect permissive or early-stage pathological processes rather than direct drivers of disability. The dissociation between pathological activity and clinical outcome highlights the importance of considering multiple biological processes when interpreting MS heterogeneity.

BRLs have emerged as a pathological marker of rapid disease progression [25], and our findings further reinforce their clinical and biological relevance. BRLs were enriched among carriers of the severity-associated SNP at the *DYSF–ZNF638* locus, suggesting a genetic contribution to chronic lesion activity. Pathologically, BRLs were associated with increased proportions of active and mixed lesions, increased microglia activation, higher BRS lesion rate, and increased cortical lesion rate. Clinically, donors with BRLs exhibited a more aggressive disease course starting at a younger age. These findings indicate that BRL associated pathology extends beyond individual lesions and is consistent with high inflammatory activity across the CNS.

Donors with cuffs and BRLs were enriched for the severity-associated common genetic variant, consistent with a previous observation linking this locus to prolonged inflammation and chronic lesion activity [25]. While these associations are observational and mechanistic interpretations remain speculative, this variant has been associated with increased neuro-axonal loss in MS [5, 13, 19, 35], suggesting increased immune activation associating with genetically higher susceptibility to neuro-axonal damage [41]. Mechanistically, this locus has been linked to altered regulation of flanking gene *ZNF638*, encoding NP220, a component of the HUSH complex involved in transcriptional silencing of viral DNA, including EBV-derived sequences [13]. Reduced suppression of latent EBV activity could enhance antigen presentation and may contribute to sustained compartmentalized immune activation, potentially supporting chronic lesion activity rather than directly determining disease susceptibility. In addition, the second flanking gene, *DYSF*, encodes dysferlin, a mediator of membrane repair following cellular injury [12, 21, 36]. Dysferlin expression is increased in normal-appearing grey matter in MS [13], and impaired dysferlin-mediated repair mechanisms may further contribute to persistence of tissue damage within chronically inflamed lesions.

Remyelination efficiency captures donor-specific repair capacity [7] and reflects another process that can contribute to inter-individual variability. In contrast to inflammatory features, remyelination efficiency was not associated with the genetic variants examined, suggesting that repair capacity may be more strongly influenced by local tissue environment and cumulative disease burden. Poor remyelination efficiency aligned with a pathological profile dominated by mixed and inactive lesions, increased BRS- and cortical lesion rate, and the dimension associated with gliosis and chronic scar formation. Clinically, these donors exhibited a less favorable disease course. These findings support the idea that a less permissive tissue environment, marked by ongoing inflammation and gliosis, may limit effective remyelination.

In contrast to donor-specific pathological features, the presence of active lesions primarily reflects a transient inflammatory disease state, associated with ongoing relapse-related activity [32]. Although active lesions showed overlapping pathological and clinical associations with several donor-specific features, the donor-level features described here likely reflect broader and more persistent biological processes that shape lesion evolution across the CNS. While these features define subgroups, they frequently co-occur, reflecting overlapping pathological processes that may together influence lesion development and persistence within individual donors. Donors lacking all four features did not exhibit an entirely benign disease course, underscoring the multifactorial nature of MS.

Our findings on post-mortem tissue raise important questions regarding the translation of these pathological features into clinical practice. If identifiable, ante-mortem donor-specific features such as perivascular cuffs, BRLs, and remyelination capacity could help predict disease course and guide personalized therapeutic strategies targeting distinct biological pathways. Achieving this will require reliable in vivo biomarkers. Recent advances in imaging, including translocator protein 18 kDa positron emission tomography (TSPO-PET), have demonstrated that BRLs can be detected in patients with MS [25]. Detection of BRLs using routinely applied magnetic resonance imaging (MRI) techniques would further enhance their clinical applicability. In contrast, visualization of microglial nodules and perivascular cuffs will likely require imaging modalities with higher spatial resolution. Although remyelination can also be assessed using MRI and PET [38], these methods currently provide indirect measures of myelin repair. The identification of imaging, fluid, or extracellular vesicle biomarkers associated with these pathological features may therefore facilitate longitudinal studies into their temporal dynamics and biological significance. Candidate biomarkers include established markers of axonal injury, myelin damage, and astrocyte activation, including NFL, MOG, and GFAP [1, 41], as well as molecules that have been previously associated to pathological processes, including CHI3L1, OPN, and C1Q in nodules, CHIT-1 and GPNMB in mixed lesions, and TGFB, EGF, and IGF1 in remyelinating environments [7, 23, 43].

Beyond the four donor-specific pathological features analyzed here, additional variables may further refine our understanding of inter-individual heterogeneity in MS pathology. For example, iron-containing lesions identified using Turnbull blue staining are clinically relevant, as their in vivo correlates, paramagnetic rim lesions on MRI, are associated with progression independent of relapse activity [46]. However, Turnbull blue staining is not routinely performed within the NBB cohort and was therefore not included in the present analyses. Moreover, lesion sampling may be biased toward white matter pathology, as white matter lesions are more readily identified macroscopically and by MRI than grey matter lesions. In addition, remyelination efficiency likely differs between white and grey matter, whereas cortical remyelination remains challenging to assess. While MRI-guided tissue sampling was available for the majority of donors, variability in sampling procedures may still have modestly favored detection of active lesions in some cases. Future studies integrating broader genetic datasets may further clarify the contribution of genetic variation to pathological heterogeneity beyond the two SNPs studied here. Finally, quantification of total lesion burden measured by area affected and spatial lesion distribution may provide additional clinically relevant dimensions of pathology.

In conclusion, this study demonstrates that perivascular cuffs, microglial nodules, BRLs, and remyelination efficiency reflect biologically relevant pathological processes that vary between individuals with MS. These donor-specific features provide insight into how disease biology is expressed at the individual level and help contextualize variability in lesion type distribution and clinical trajectories. Integrating donor-specific pathology with genetic background, lesion classification, and clinical data, offers a more biologically grounded framework for interpreting post-mortem MS tissue and studying disease heterogeneity. Such approaches may improve the design and interpretation of future neuropathological and translational studies.

## Supplementary Information

Below is the link to the electronic supplementary material.

Supplementary file1 (PDF 1410 KB)

Supplementary file2 (XLSX 23 KB)
