# Supplementary material for: Donor-specific pathological features associate with genetic background, lesion type distribution, and clinical heterogeneity in multiple sclerosis
Source: Acta Neuropathol. 2026 Jun 25;151(1):72. doi: 10.1007/s00401-026-03040-3 (PMC13303759; doi:10.1007/s00401-026-03040-3)
Supplement: Supplementary file 1 — Supplementary file1 (PDF 1410 KB) [file 401_2026_3040_MOESM1_ESM.pdf]

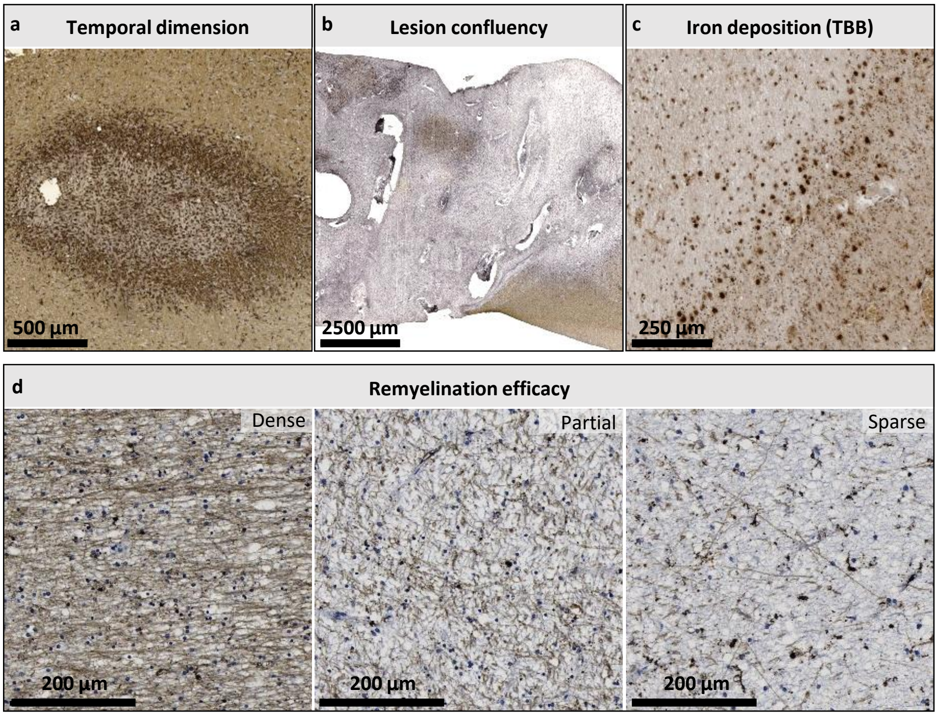

Supplementary Fig 1. Additional lesion morphologies. a) A lesion displaying properties of an active lesion (hypercellular center) and mixed lesion (microglia/macrophage accumulation along the edges). b) A confluent lesion containing myelin patches in otherwise demyelinated areas. c) Iron deposition at the rim of a mixed lesion. d) Remyelinated lesions with distinct degree of remyelination. A, b and d are stained for HLA (black) and PLP (brown). C is stained for iron (Turnbull Blue).

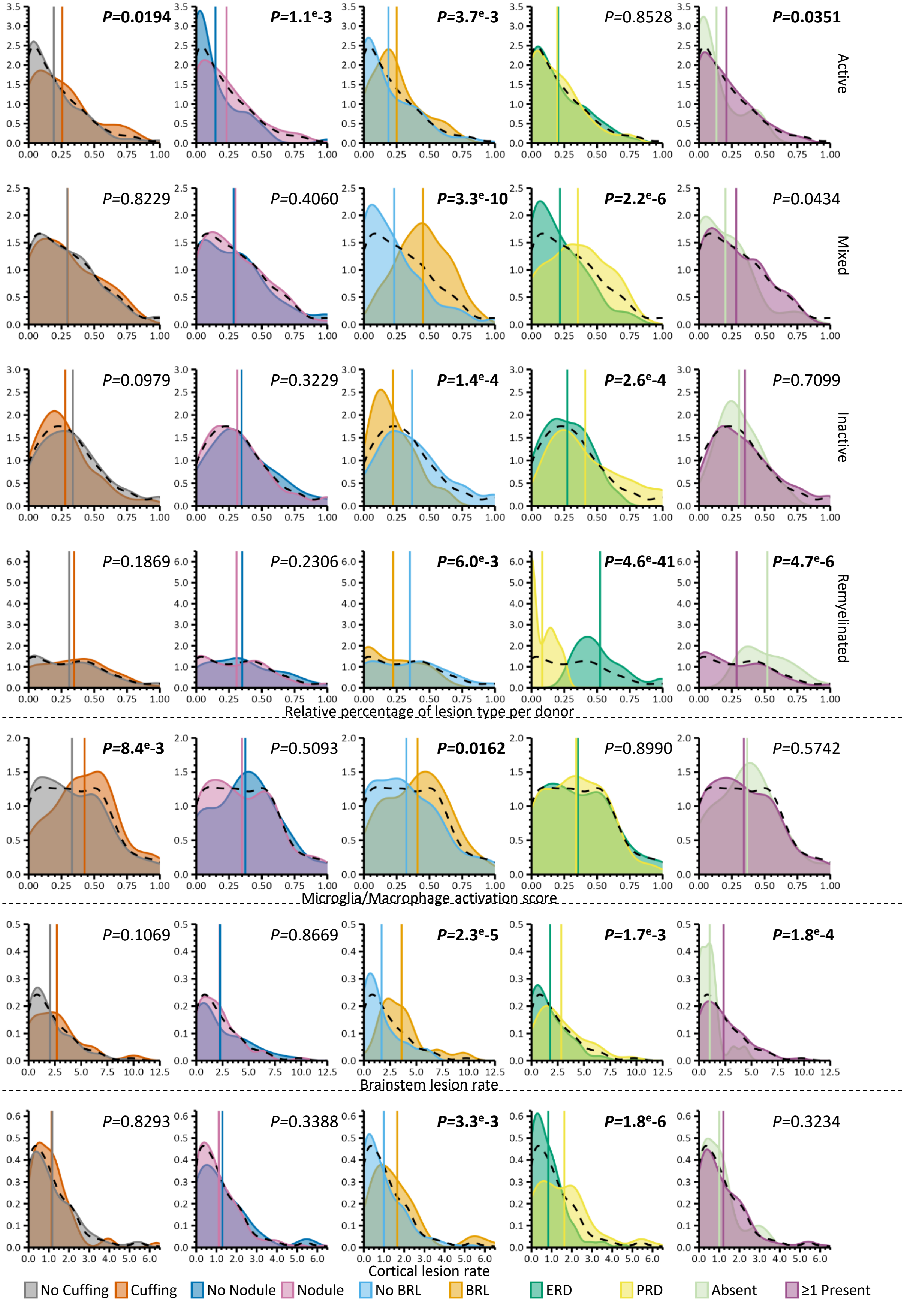

Supplementary Fig 2. Distribution of pathological features across distinct donor cohorts. Density plots of pathological parameters after stratification for donor-specific pathologies. Displayed *P*-values are uncorrected, highlighted *P*-values stay significant after FDR correction per tested parameter.

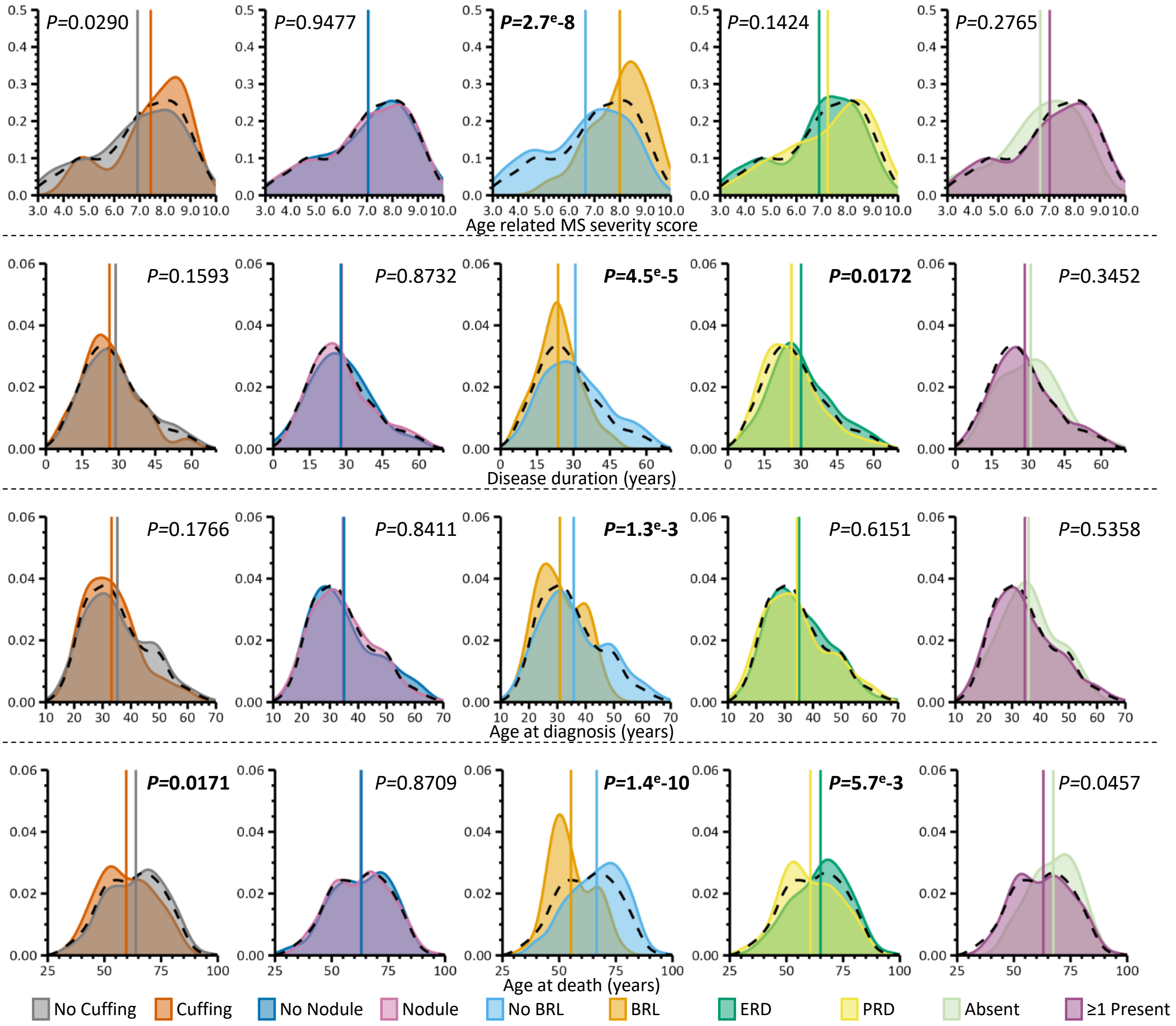

Supplementary Fig 3. Distribution of clinical features across distinct donor cohorts. Density plots of clinical parameters after stratification for donor-specific pathologies. Displayed *P*-values are uncorrected, highlighted *P*-values stay significant after FDR correction per tested parameter.
